# Genomic integration of unclonable gene expression cassettes in *Saccharomyces cerevisiae* using rapid cloning‐free workflows

**DOI:** 10.1002/mbo3.978

**Published:** 2020-01-15

**Authors:** Vicente F. Cataldo, Valeria Salgado, Pedro A. Saa, Eduardo Agosin

**Affiliations:** ^1^ Department of Chemical and Bioprocess Engineering Pontificia Universidad Católica de Chile Santiago Chile

**Keywords:** cloning‐free, DNA Assembly, gene integration, *Saccharomyces cerevisiae*, toxic genes, yeast

## Abstract

Most DNA assembly methods require bacterial amplification steps, which restrict its application to genes that can be cloned in the bacterial host without significant toxic effects. However, genes that cannot be cloned in bacteria do not necessarily exert toxic effects on the final host. In order to tackle this issue, we adapted two DNA assembly workflows for rapid, cloning‐free construction and genomic integration of expression cassettes in *Saccharomyces cerevisiae*. One method is based on a modified Gibson assembly, while the other relies on a direct assembly and integration of linear PCR products by yeast homologous recombination. The methods require few simple experimental steps, and their performance was evaluated for the assembly and integration of unclonable zeaxanthin epoxidase expression cassettes in yeast. Results showed that up to 95% integration efficiency can be reached with minimal experimental effort. The presented workflows can be employed as rapid gene integration tools for yeast, especially tailored for integrating unclonable genes.

## Graphical abstract

We adapted two DNA assembly workflows for rapid, cloning‐free construction and genomic integration of expression cassettes in *Saccharomyces cerevisiae*. The methods require few simple experimental steps, and their performance was evaluated for the assembly and integration of unclonable zeaxanthin epoxidase expression cassettes. Results showed that up to 95% integration efficiency can be reached with minimal experimental effort.

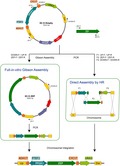

## 1 INTRODUCTION

Efficient DNA assembling and cloning methods are critical for the success of metabolic engineering applications and progress of synthetic biology. In spite of the advances in chemical DNA synthesis technology, assembly methods are still required for the construction of large DNA fragments, that is, >200 bp (Hughes & Ellington, 2017; Kosuri & Church, 2014). For these tasks, there are currently several commercial and in‐house in vitro DNA assembly technologies available to genetic engineers. Depending on the scientific principle underpinning the assembly technology, two types of techniques can be distinguished: restriction/ligation‐based (e.g., Biobrick (Shetty, Endy, & Knight, 2008) and Golden Gate (Engler, Gruetzner, Kandzia, & Marillonnet, 2009; Engler, Kandzia, & Marillonnet, 2008)), and sequence homology‐based methods. Due to its high versatility and assembly efficiency, the latter has gained more acceptance in the field (Chao, Yuan, & Zhao, 2015).

The first assembly method based on sequence homology was OE‐PCR (Overlap Extension PCR; Horton, Hunt, Ho, Pullen, & Pease, 1989). This ligase‐free approach assembles DNA fragments in two rounds of PCR. First, DNA templates are separately amplified using primers to yield overlapping regions. The products are then mixed in a second round of PCR where overlapping regions act as primers. Finally, DNA polymerase extends and produces the sought‐spliced product. Although this technique is still widely used, it is laborious and has been gradually replaced by more efficient assembly methods like SLIC (Li & Elledge, 2007), USER (Bitinaite et al., 2007; Vaisvila & Bitinaite, 2013), and Gibson assembly (Gibson et al., 2009). Particularly, the latter has been widely adopted in the community because of its simplicity for joining multiple DNA parts in a single isothermal reaction. As in OE‐PCR, Gibson assembly employs overlapping PCR products, but in this case, a T5 exonuclease is used to hydrolyze 5′ ends, thereby generating complementary overhangs for specific annealing. Lastly, the DNA polymerase and Taq ligase sequentially repair the double strand yielding a covalently joined seamless product.

Another group of efficient assembly methods exploits the inherent homologous recombination (HR) machinery of *Saccharomyces cerevisiae*. HR assembly in *S. cerevisiae* was first reported for the construction of yeast extrachromosomal expression vectors (Juhas & Ajioka, 2017; Ma, Kunes, Schatz, & Botstein, 1987; Oldenburg, Vo, Michaelis, & Paddon, 1997; Raymond, Pownder, & Sexson, 1999), but has also been extended for the construction of expression vectors for other model organisms (Dudley et al., 2009; Joska, Mashruwala, Boyd, & Belden, 2014; Kilaru & Steinberg, 2015). This method requires insertion of homology regions by PCR to both the target DNA parts (e.g., gene, markers, etc) and the linearized backbone vector. The PCR products are then directly transformed in yeast where the circular vector is assembled by HR. Using this approach, an assembly of up to nine fragments in a 21‐kb vector was carried out using 60‐bp overlap regions (Kuijpers et al., 2013). Furthermore, so far this method has been shown to be much more effective for dealing with large DNA fragments than traditional cloning in *Escherichia coli* (Kouprina & Larionov, 2016). For instance, due to instability issues of large DNA constructs in *E. coli*, in vivo DNA assembly in yeast was critical for assembling the first synthetic bacterial genomes (Gibson, Benders, Andrews‐Pfannkoch, et al., 2008; Gibson, Benders, Axelrod, et al., 2008; Gibson et al., 2010).

Besides its use as DNA assembly tool and owed to its well‐studied and highly tunable genetics, yeast is currently considered a model organism for biotechnological applications (Lian, Mishra, & Zhao, 2018). Shuttle vectors are plasmids commonly used for gene expression in *S. cerevisiae* (Gnügge & Rudolf, 2017). These plasmids have genetic sequences that enable their maintenance in both *E. coli* and *S. cerevisiae*. This feature enables the construction (usually using in vitro assembly methods), analysis, and amplification of the plasmid in *E. coli* for subsequent yeast transformation. However, some genes are unclonable in *E. coli* even in the absence of a bacterial promoter, pointing to DNA toxicity and/or genetic instability (Kimelman et al., 2012). Examples of known genes that cannot be cloned in *E. coli* include Vssc1 sodium (Lee & Soderlund, 2009) and Cch1 calcium (Vu, Bautos, Hong, & Gelli, 2013) channels, to name a few. In yeast, extrachromosomal expression vectors can be directly assembled by in vivo recombination, thereby bypassing bacterial transformation. However, this approach has been less explored for direct assembly of integrative constructs. Interestingly, Shao, Zhao, & Zhao (2009) evaluated and demonstrated a high capacity of yeast for assembling and integrating functional expression constructs from PCR‐amplified fragments in a single transformation event. These results motivated us to develop simpler assembly and integration workflows for yeast that avoid bacterial transformation altogether.

In this work, we report two simple and rapid workflows for direct assembly and site‐specific integration of gene expression cassettes in *S. cerevisiae* without bacterial cloning steps. One method is based on a modification of Gibson assembly —here termed full in vitro Gibson assembly—whereas the other is based on a direct assembly of PCR‐amplified fragments by yeast HR. The methods were validated for the construction and high efficiency integration of expression cassettes for *Haematococcus lacustris* and *Solanum lycopersicum* zeaxanthin epoxidase (ZEP) genes. These genes cannot be cloned in *E. coli* due to toxic effects. The presented workflows provide simple, rapid, and efficient gene assembly and integration alternatives for yeast, especially suitable but not limited to unclonable genes.

## 2 MATERIALS AND METHODS

### 2.1 Strains, growth conditions, and DNA templates

Codon‐optimized ZEP genes from *H. lacustris* (HlZEP) and *S. lycopersicum* (SlZEP) were synthesized by Genscript. Full gene sequences can be found in Table A1. The XI‐5 integrative vector with bidirectional PGK1/TEF1 promoters was constructed using the plasmid set described by Mikkelsen et al. (2012). *Saccharomyces cerevisiae* BY4742 strain was used in all transformations. Cultures were grown in complete YPD medium (20 g/L of peptone, 20 g/L of glucose, and 10 g/L of yeast extract) at 30°C. Yeast transformants were incubated in synthetic medium plates containing: 1.8 g/L of yeast nitrogen base, 5 g/L of ammonium sulfate, 0.8 g/L of CSM‐Ura mixture (Sunrise Science Products), 20 g/L of glucose, and 20 g/L of agar.

### 2.2 DNA construction and assembly

**Figure 1:**
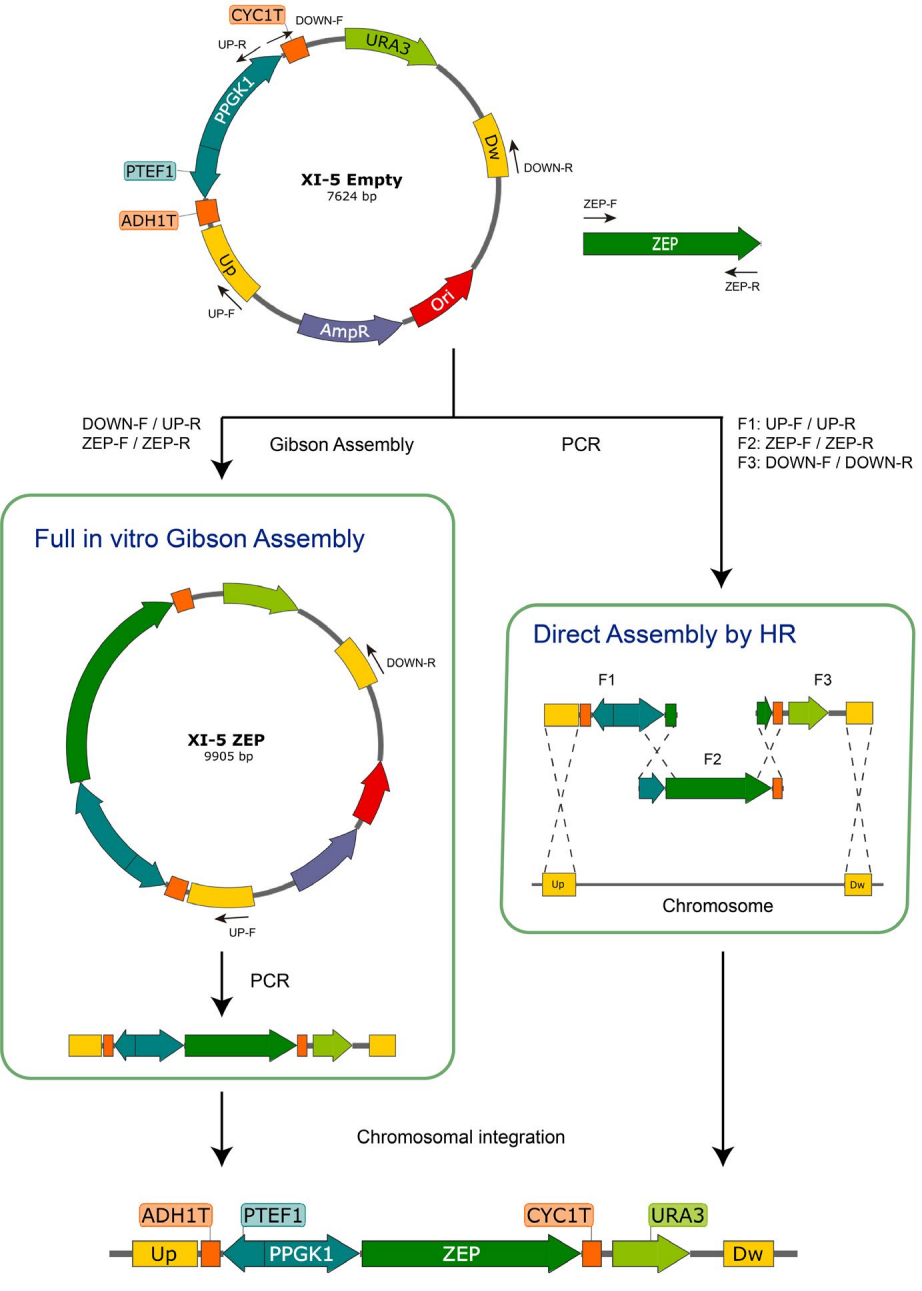
Schematic overview of cloning‐free methods for assembly and integration of expression cassettes exemplified for ZEP expression constructs. Full in vitro Gibson assembly bypasses bacterial amplification using the reaction product as a template for PCR amplification of the desired integration cassette. Direct assembly by HR is based on the transformation of linear overlapping PCR products which are assembled and integrated into the genome in a single transformation event. The primers used in each method are indicated next to the arrows. F1, F2, and F3 refer to fragments 1, 2, and 3

**Figure 2:**
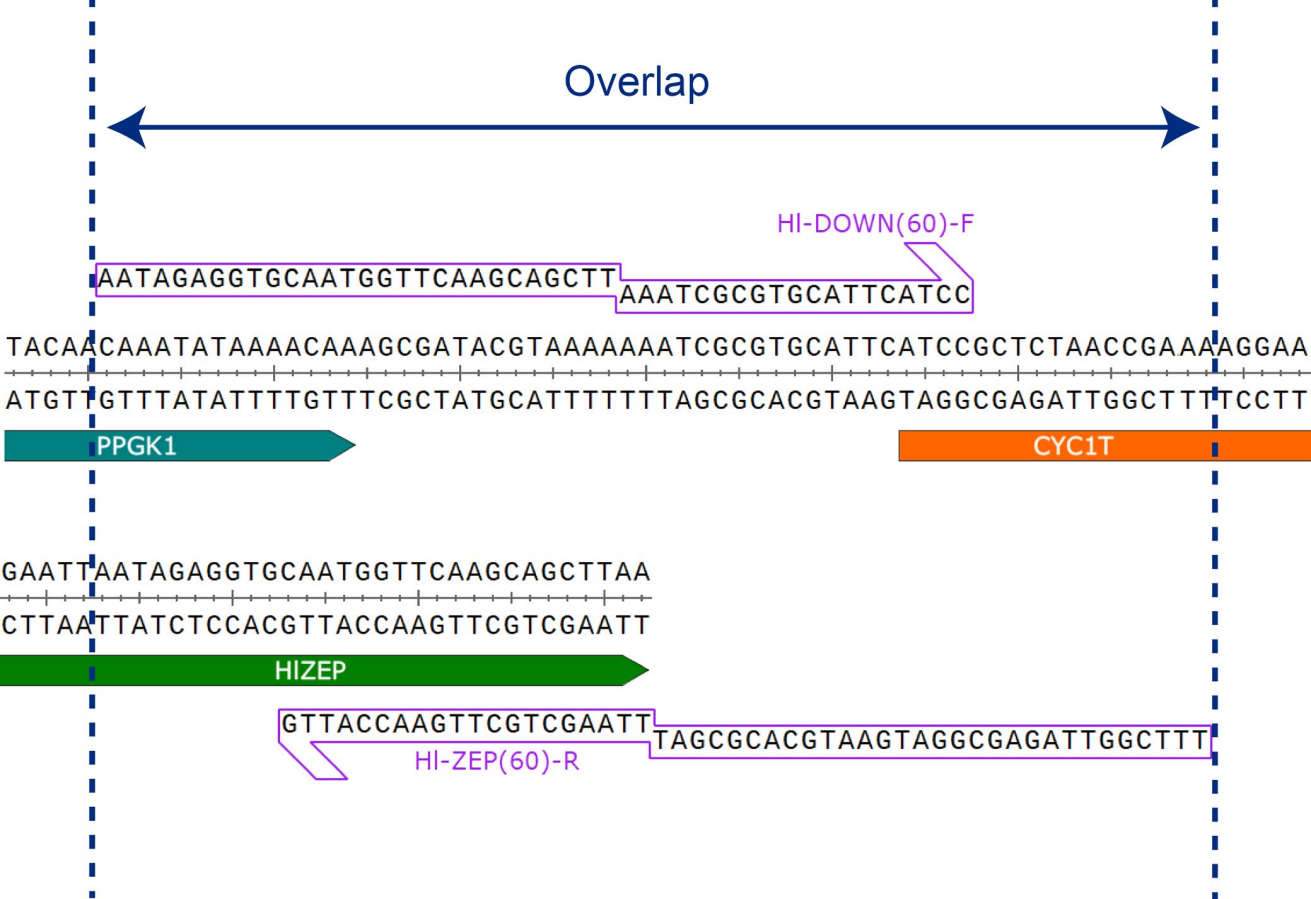
Illustration of primer design for generation of overlapping fragments. The homology region between fragments is included in the 5′ nonpriming sequences of the primers

Direct assembly of expression cassettes by HR was performed using three PCR‐amplified fragments (F1, F2, and F3, Figure 1). To generate each set of fragments, six primers were designed: UP‐F, UP‐R, DOWN‐F, DOWN‐R, ZEP‐F, and ZEP‐R. Overlapping regions between fragments were included in the 5′ sequence of the primers (exemplified in Figure 2). To evaluate the effect of the overlap length on the assembly efficiency, three sets of primers were designed for each HlZEP and SlZEP expression cassette with overlap lengths of 40, 60, and 100 bp. Fragments for Gibson assembly were generated with the same primers used for the 40 bp homologous recombination cassettes: DOWN‐F/UP‐R and ZEP‐F/ZEP‐R. Primer sequences are listed in Table A2.

DNA fragments for both assembly methods were amplified by PCR using Phusion High‐Fidelity DNA Polymerase (Thermo Fisher Scientific). PCR reactions were carried out in 100 µl containing 0.5 pmol/μl of each primer, HF buffer 5×, and 0.02 U/μl of Phusion DNA polymerase. The PCR protocol consisted of an initial denaturation at 98°C for 2 min, then 35 cycles of amplification (98°C for 10 s, 60°C for 30 s, and 72°C for 3 to 6 min), followed by a final extension of 72°C for 10 min. PCR products were purified by gel extraction using Wizard SV Gel and PCR Clean‐Up kit (Promega), according to the manufacturer's instructions.

Gibson assembly was performed using a master mix composed of T5 exonuclease 1 U/μl (NEB), Phusion DNA polymerase 2 U/μl, Taq DNA ligase 40 U/μl (NEB), and 5× isothermal buffer. The latter contained PEG‐8000 (25%), Tris‐HCl pH 7.5 (500 mM), MgCl_2_ (50 mM), DTT (50 mM), dATP, dTTP, dCTP, dGTP (1 mM), and NAD^+^ (5 mM). Assembly was achieved by mixing 2.5 µl containing 100 ng of equimolar fragments with 7.5 µl of master mix and incubated at 50°C for 2 hr. Finally, 4 μl of the reaction products was used as PCR templates where the UP‐F and DOWN‐R primers were employed for the amplification of the UP‐DOWN cassettes. These PCR products were digested with 5 U of DpnI for 1 hr to eliminate the residual parental vector.

### 2.3 Yeast transformation

Transformations were performed by LiAc/SS carrier DNA/PEG method (Gietz & Schiestl, 2007) with a slight modification. In order to increase the volume of DNA fragments, a more diluted transformation mix was employed (0.09 M of lithium acetate). In the case of assembly by HR, transformations were performed using 3 pmol of each fragment mixed to a 100‐μl final volume. In full in vitro Gibson assemblies, all the resulting PCR product (~1.2 pmol in 100 μl) was used for the transformation. The transformed cells were plated on SC‐Ura agar and incubated for two to three days at 30°C. The XI‐5 empty vector linearized by SwaI digestion was used as transformation control.

### 2.4 Evaluation of integration efficiency

In each transformation, ten colonies were individually picked and cultured for 16 hr at 30°C in liquid YPD media. Genomic DNAs were then extracted using Wizard Genomic DNA Purification Kit (Promega). Confirmation of correct chromosomal integration of the assembled expression cassettes was carried out by PCR amplification of the previously extracted DNA. Four PCR rounds were performed on each strain for efficiency analysis: one that amplified the entire assembled cassette from the UP to DOWN region and three that amplified between the recombination regions of each fragment. PCR reactions were carried out with Phusion High‐Fidelity DNA Polymerase using the same cycling parameter described in section 2.2. The list of used primers can be found in Table A2.

## 3 RESULTS AND DISCUSSION

We could not clone HlZEP and SlZEP genes in the yeast integrative vector by traditional Gibson assembly regardless of the *E. coli* strain evaluated (TOP10, DH5α, and K12). This result suggests high toxicity or instability of the ZEP expression cassettes in *E. coli*. As previously reported, many gene products, either noncoding RNA or proteins, can be toxic in *E. coli* (Kimelman et al., 2012). Although in this study ZEP genes were cloned under the control of yeast PGK1 promoter, some eukaryotic promoters can still drive gene expression in *E. coli* (Antonucci, Wen, & Rutter, 1989; Gognies, Bahkali, Moslem, & Belarbi, 2012). Thus, ZEP genes may have been expressed in the transformed cells causing toxicity. Another plausible cause is related to the toxicity of the DNA itself (Kouprina & Larionov, 2016). Unclonable noncoding DNA regions have been suggested to exert such effect, but the underpinning molecular mechanisms have not been fully elucidated. Some cloned sequences can seemingly cause toxicity due to their high capacity to recruit and titrate essential DNA binding proteins such as replicator initiator DnaA (Kimelman et al., 2012) or RNA polymerase (Lamberte et al., 2017).

To tackle the above limitations, we developed two strategies that enable assembly and chromosomal integration of expression constructs in yeast without the need of bacterial transformation. As a proof of concept, the ZEP genes were used in this study. Our approaches employ the set of plasmids designed by Mikkelsen et al. (2012) as transcriptional backbones. Briefly, these vectors enable the integration of one or two genes controlled by a bidirectional promoter in specific chromosomal sites. The vector set uses URA3 as a selectable marker, which is flanked by a direct repeat to enable marker recycling and more transformation rounds. As illustrated in Figure 1, we assembled the ZEP genes into expression cassettes using the vector XI‐5 as a backbone (i.e., integration in site 5 of chromosome XI) by two different strategies: full in vitro Gibson assembly and direct assembly by HR. Both methods are PCR‐based and do not need bacterial transformation nor plasmid isolation steps.

### 3.1 Full in vitro Gibson assembly

**Figure 3:**
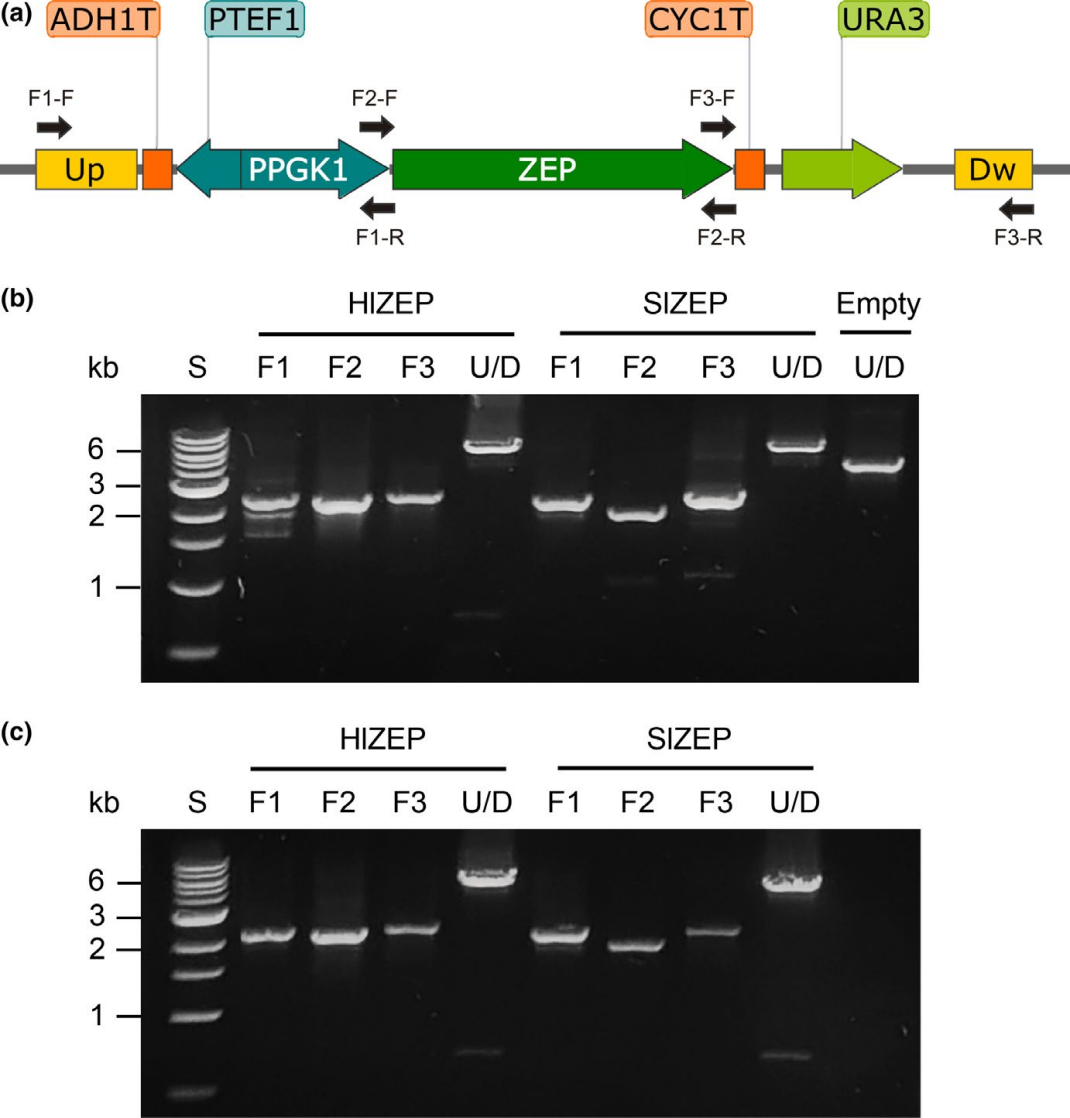
Assembly and integration of ZEP expression cassettes. (a) Scheme of assembled ZEP expression cassette with the corresponding verification primers. Genomic PCR analysis of the integrated HlZEP and SlZEP constructs for full‐in‐vitro Gibson assembly (b) and direct assembly by HR (c). S: 1 kb DNA ladder. F1, F2, and F3 refer to Fragments 1, 2, and 3, and U/D represents the UP/DOWN region. Empty refers to the backbone vector linearized by SwaI digestion. Expected PCR products (bp): Hl‐F1 (2364), Hl‐F2 (2300), Hl‐F3 (2428), Hl‐U/D (7043), Sl‐F1 (2371), Sl‐F2 (2041), Sl‐F3 (2428), Sl‐U/D (6791), Empty‐U/D (4781)

Gibson assembly requires one reaction to join DNA fragments into a vector. Typically, the reaction product is transformed and amplified in *E. coli*. For shuttle integrative vectors in yeast, like as XI‐5 (Figure 1), additional digestion with SwaI and gel purification are necessary steps for isolating the desired integrating DNA fragment and discarding bacterial elements (Ori and Amp). Here, we propose a simple modification of this protocol, where the Gibson assembly product is used as DNA template in a PCR reaction that amplifies only the segment that will be integrated into the yeast genome (Figure 1). This PCR reaction can be transformed directly after digestion with Dpn1 (to eliminate the parental vector), without subsequent clean‐up steps. Thus, both the *assembly and amplification* of the Gibson assembly product occur in vitro, as opposed to the conventional Gibson method where the assembly takes place in vitro and the amplification occurs in *E. coli*. This method was applied to integrate HlZEP and SlZEP expression cassettes in yeast. Resulting colonies were screened for successful integration by genomic PCR with a set of primers that amplified three segments and the whole integrated construct (Figure 3). As shown in Table 1, a 95% chromosomal integration efficiency was achieved for HlZEP and SlZEP expression cassettes, slightly less than that for SwaI digestion of the empty vector (100%). Notably, this method is likely limited by the length of the integrating fragment that can be amplified by high‐fidelity polymerases. However, for integrative cassettes of one or two genes (5–8 kb), one PCR reaction using high‐fidelity polymerases can easily render the required DNA amount (1 pmol) for efficient yeast transformation.

**Table 1:** Integration efficiencies of assembled ZEP expression constructs based on PCR analysis of genomic DNA of 20 colonies (10 for HlZEP and 10 for SlZEP)

	Integration efficiency (%)
Digested empty vector	100
Full in vitro Gibson assembly	95
Direct assembly by HR—40 bp overlap	20
Direct assembly by HR—60 bp overlap	50
Direct assembly by HR—100 bp overlap	85

### 3.2 Direct assembly by homologous recombination

**Figure A1:**
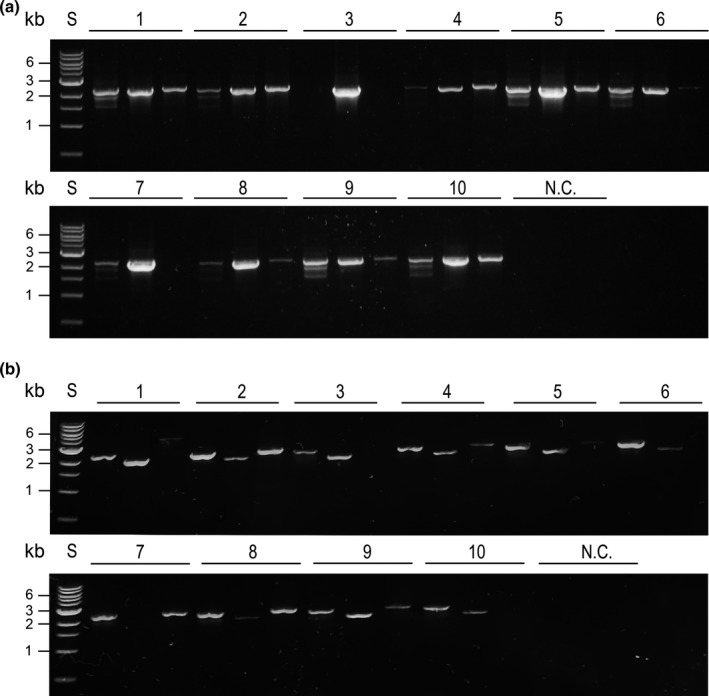
Examples of PCR analysis of genomic DNA of 10 yeast transformants obtained for direct assembly by HR of (a) HlZEP with 100 bp of overlap, and (b) SlZEP with 60 bp of overlap. PCR of fragments 1, 2, and 3 (refer to Figure 3) is shown for each transformant (1–10). S: 1 kb DNA ladder. N.C.: Negative control

Based on the recombination scheme proposed by Shao et al. (2009), we developed a direct in vivo DNA assembly and site‐specific integration method from linear PCR products. Three overlapping fragments were generated by PCR using the backbone vector and the gene of interest as templates (Figure 1). Similar to Gibson assembly, primers were designed with a nonpriming sequence at the 5′ end that is homologous to the 5′ end of the fragment to join (exemplified in Figure 2). All fragments were cotransformed in yeast, which assembled and ultimately integrated the construct by HR. To evaluate the effect of the overlap length on the assembly, we transformed each HlZEP and SlZEP construct with a set of fragments with 40, 60, and 100 bp of homology. Correct assembly and integration were verified by genomic PCR analysis of transformants using primers that annealed specifically in the overlap regions (Figure 3). Examples of the analysis of more colonies can be found in Figure A1. The overlap length had a strong positive effect on the assembly and integration efficiency, reaching up to 85% efficiency when a 100 bp overlap segment was employed (Table 1). Notably, direct assembly by HR requires only one PCR round (full in vitro Gibson assembly requires two), and thus can be readily performed in a day.

The proposed methodology for direct assembly and integration of expression cassettes by HR was adapted from the so‐called DNA assembler method presented by Shao et al. (2009), with the incorporation of some important features for streamlining the workflow. In the DNA assembler method, promoter‐gene‐terminator units are assembled by OE‐PCR, while the helper fragment—which contains the marker and integration site—is obtained by enzyme digestion. Since all these elements are included in the backbone vector in this method, additional in vitro steps aside of the PCR amplification of the designed fragments are unnecessary. Moreover, here we demonstrated effective in vivo assembly with single specific integration site, instead of repeated δ sites as in (Shao et al., 2009). The proposed method avoids multiple integrations events that usually occur in δ sites (Sakai, Shimizu, & Hishinuma, 1990; Wang, Wang, & Da Silva, 1996), allowing finer control of the gene copy number. Finally, instead of single homology arm integration, we proposed a double crossing‐over configuration, which avoids direct repeats sequences and increases the genomic stability of the construct (Gnügge & Rudolf, 2017; Taxis & Knop, 2006).

As mentioned earlier, yeast recombination cloning has been extensively reported for the construction of yeast extrachromosomal vectors or plasmids for other species. However, this approach is futile when the goal is to integrate expression cassettes, as extrachromosomal replicating elements (CEN/ARS, 2µ) prevent chromosomal integration. To overcome this obstacle, Chou, Patel, & Gartenberg (2015) constructed a series of conditional shuttle vectors where the CEN/ARS elements were flanked by loxP sites. This feature enabled elimination of the replicating sequences when the vectors were transformed in Cre recombinase‐expressing bacteria. In this way, the plasmid can be assembled extrachromosomally in yeast and converted to an integrative vector in bacteria, which can be then used to transform the yeast again. This time‐consuming cloning strategy requires several transformations and plasmid isolation steps. In contrast, the direct assembly by HR proposed here simplifies the assembly and integration of expression cassettes to only few simple steps.

## 4 CONCLUSION

In this work, we presented two simple, rapid, and effective workflows for cloning‐free assembly and integration of gene expression cassettes in *S. cerevisiae*. While both approaches are inspired on reported assembly strategies, the introduced adaptations enabled substantial reductions in experimental efforts while maintaining high integration efficiencies. The first method—termed full in vitro Gibson assembly—showed the best integration efficiency (95%), while the second—direct assembly by HR—was faster (it can be performed in a day) with a reasonably high efficiency (85%). Importantly, both techniques can be readily employed to join more than three fragments, for example, construction of bidirectional expression cassettes by a four‐fragment assembly. Although the tools presented here are particularly tailored for genes that are unclonable in *E. coli*, they can also be used as general‐purpose, rapid, and efficient gene integration alternatives methods.

## CONFLICT OF INTEREST

None declared.

## AUTHOR CONTRIBUTIONS

Vicente Cataldo Conceptualization‐Lead, Investigation‐Supporting, Methodology‐Lead, Writing‐original draft‐Lead, Writing‐review & editing‐Lead; Valeria Salgado Investigation‐Lead, Methodology‐Supporting, Writing‐original draft‐Supporting; Pedro Saa Methodology‐Supporting, Writing‐original draft‐Supporting, Writing‐review & editing‐Supporting; Eduardo Agosin Supervision‐Lead, Writing‐original draft‐Supporting

## ETHICS STATEMENT

None required.

## Data Availability

All data generated or analyzed during this study are included in this published article.

## Appendix A

**Table A1:** HlZEP and SlZEP DNA sequences

HlZEP
ATGTTGTTACATACTTCTTCATTGCCAAGATGTCAAGCTGCAGGTC ATGTTAAGTCAACAGTTTCTATCCATGTTCCAGCTTCTCCAAGATT AGTTCCATCATGTCATCATGGTTCTGCTGCACCAGTTTCACCAAG AAGATGGACTCCACCATCAGTTTCTTGTCCAGCTGTTTTGGAAGC TGCAAGACCAGGTCAACAAGAAAGATTAGAAGGTGCAGTTCCAG AATTGTGTCCAGGTTTAACTATTGTTATTGCAGGTGCTGGTATTTCT GGTTTGACATTAGCTTTGTCATTGTTGAAGAAAGGTGTTAAGTGTC AAGTTTTGGAAAGAGATTTGACAGCTATTAGAGGTGAAGGTAAA ATTAGAGGTCCAATTCAAGTTCAATCAAATGCTTTAGCTGCATTGG AAGCAATTGATCCAGTTGTTGCTGATGATATTATGGCACATGGTTG TATTACTGGTGACAGAATTAATGGTTTGTGTGATGGTGTTTCTGGT GACTGGTATGTTAAATTTGATACATTTCATCCAGCTGTTGAAAGAG GTTTGCCAGTTACTAGAGTTATTAATAGAGTTACATTGCAACAATT GTTAGCAGAAGCTGTTATTAGATTGGGTGGTGAAGATATGATTTTA GGTGGTTGTCATGTTACTGCTTATGAAGAATTTGTTGATAGAGCAT CAGGTAAACAACAAGTTGCTGCAATTTTGGAAGATGGTAGAAGA TTTGAGGGTGACTTGTTAGTTGGTACAGATGGTATTTGGTCTAAGA TCAGACAACAAATGATTGGTGACGCACCAGCTCATTACTCTGAAT ACACTTGTTACACAGGTATCTCAGAATACGTTCCAGCTGATATTGA TGTTGTTGGTTACAGAGTTTTCTTGGGTAACAGACAATACTTCGTT TCTTCAGATGTTGGTGAAGGTAGAATGCAATGGTATGCTTTTCATC AAGAACCAGCAGGTGGTCAAGATACTTTGGGTCAAAGAAAGGCT AGATTGTTGCAATTGTTCGGTCATTGGAACTACAACGTTGTTGATT TGATCAGAGCTACACCAGAAGAAGATGTTTTGAGAAGAGATATCT ATGATAGAGCACCAATTTTTAAGTGGGCTCAAGGTAGAGTTGCAT TGATGGGTGACTCTGCACATGCTATGCAACCAAATTTGGGTCAAG GTGGTTGTATGGCAATGGAAGATGCTTTTCAATTGGCAAATGATAT TGCTGCAATGGCAGAAAAAGCTGGTCAACAAGGTGCTTTAGGTC CATTGGCAGTTCAACAATGTTTGAGAAGATACCAAGATCAAAGA ATCATGAGAGTTTCTGCTATTCATGGTATGGCTGGTATGGCTGCTTT TATGGCTTCAACTTACAAAGCATATTTGGGTGAAGGTTTAGGTCCA TTGTCTTGGTTGACAAGATACAAGATCCCACATCCAGGTAGAGTT GTTGGTCAATGGGTTATGAAATTGACTATGCCAGGTGTTTTGGGTT GGGTTTTAGGTGGTAATACAGATAAATTGGAAGCTGCAAGAGCTC CACATTGTAGATTGTCTGATAAGCCAAGATGTTTCCAAGAATCAG AATTTGAATTGTTGATGAGAGATGATGATTTGTTAGCTGAAAGAG CAAATGCTGATTGGTTGTTAGTTGCTGAAAGATTGGCAAGACCAC CAACTGCTTTAAATGCTGCACAAGGTCAAGGTCAACATGTTTACG CATTGGCTATGATGGATACATTAGTTCCAGGTTCAGGTTCTTCATC TTCATCTGGTGGTTCATCTTTTCCATTGGCTGCAGCTGGCATGTCT AGAGCTGAAGAAGAAGGTGTTACTTTGCCAAGACCAGGTGGTTT CGGTTTAGCACCATCAGAATACAAAGGTGTTTATTTGAATCCAGC ACCAGAAGCTACTCCAGCAGCTGAACCAGGTGTTACATTAGTTGG TAGATCACCATCTTGTCATTTGGTTTTGGATAATCCATCTTGTGCTG AACAACATGCAAGAATTGAAATGCAATCTGCTGGTAGATACTTCG CACATGATTTGGGTTCAAACAATGGTACATGGGTTAACGGTCATA GATTGGAAAAGGGTGAAAGAGCTATGTTGCATCCAGGTGACGTTT TAAGATTTGGTAGACAAGGTTCTGAAGTTTTTACTGTTAAATTGCA ACATACATCATACAGAAATGCTGAAGTTAGAGGTGACTGTTACCA AAGAATTAATAGAGGTGCAATGGTTCAAGCAGCTTAA
SlZEP
ATGTACTCTACTGTTTTCTATACATCAGTTCATCCATCTACTTCAGT TTTGTCAAGAAAGCAATTGCCATTGTTAATTTCTAAGGATTTCTCA GCTGAATTGTACCATTCTTTGCCATGTAGATCATTAGAAAACGGTC ATATCAATAAGGTTAAGGGTGTTAAGGTTAAGGCTACTATCGCTGA AGCACCAGTTACTCCAACAGAAAAGACTGATTCTGGTGCAAATG GTGACTTGAAAGTTCCACAAAAGAAATTGAAGGTTTTGGTTGCTG GTGGTGGTATTGGTGGTTTAGTTTTTGCATTGGCTGCTAAGAAAAG AGGTTTCGATGTTTTGGTTTTCGAAAGAGATTTGTCTGCTATTAGA GGTGAAGGTCAATACAGAGGTCCAATTCAAATTCAATCAAATGCT TTGGCTGCATTAGAAGCAATCGATTTGGATGTTGCTGAAGATATTA TGAATGCAGGTTGTATCACAGGTCAAAGAATTAATGGTTTGGTTG ATGGTATTTCTGGTAACTGGTACTGTAAGTTCGATACTTTTACACC AGCTGTTGAAAGAGGTTTGCCAGTTACTAGAGTTATTTCAAGAAT GACATTGCAACAAATCTTGGCTAGAGCAGTTGGTGAAGAAATCAT CATGAACGAATCAAACGTTGTTGATTTCGAAGATGATGGTGAAAA GGTTACTGTTGTTTTAGAAAACGGTCAAAGATTCACTGGTGACTT GTTAGTTGGTGCTGATGGTATTAGATCTAAAGTTAGAACTAATTTG TTTGGTCCATCTGAAGCTACATATTCAGGTTACACTTGTTATACAG GTATTGCTGATTTTGTTCCAGCAGATATTGATACTGTTGGTTACAG AGTTTTCTTGGGTCATAAGCAATACTTCGTTTCTTCAGATGTTGGT GGTGGTAAAATGCAATGGTACGCTTTCTACAACGAACCAGCAGGT GGTGCTGATGCACCAAACGGTAAAAAGGAAAGATTGTTGAAGAT CTTCGGTGGTTGGTGTGATAACGTTATCGATTTGTTGGTTGCTACA GATGAAGATGCAATCTTGAGAAGAGATATATATGATAGACCACCA ACTTTTTCTTGGGGTAGAGGTAGAGTTACATTGTTGGGTGACTCA GTTCATGCTATGCAACCAAATTTGGGTCAAGGTGGTTGTATGGCTA TTGAAGATTCTTACCAATTAGCATTGGAATTAGAAAAAGCATGTT CAAGATCAGCAGAATTTGGTTCACCAGTTGATATTATTTCTTCATT AAGATCTTATGAATCAGCTAGAAAATTGAGAGTTGGTGTTATTCAT GGTTTGGCAAGAATGGCTGCAATCATGGCTTCTACTTACAAAGCA TATTTGGGTGTTGGTTTGGGTCCATTATCATTTTTGACACAATACA GAATACCACATCCAGGTAGAGTTGGTGGTAGAGTTTTTATTGATTT GGGTATGCCATTGATGTTATCTTGGGTTTTAGGTGGTAATGGTGAC AAATTGGAAGGTAGAATTAAACATTGTAGATTATCAGAAAAGGCT AACGATCAATTGAGAAAGTGGTTCGAAGATGATGATGCATTGGAA AGAGCTACTGATGCAGAATGGTTGTTATTGCCAGCTGGTAATGGTT CTTCAGGTTTAGAAGCAATTGTTTTGTCAAGAGATGAAGATGTTC CATGTACTGTTGGTTCTATTTCACATACAAACATCCCTGGTAAATC AATCGTTTTGCCATTACCACAAGTTTCTGAAATGCATGCTAGAATT TCATGTAAAGATGGTGCTTTCTTTGTTACTGATTTGAGATCTGAAC ATGGTACTTGGGTTACAGATAACGAAGGTAGAAGATATAGAACTT CACCAAATTTTCCAACAAGATTCCATCCATCTGATGTTATCGAATT TGGTTCAGATAAAGCTGCTTTTAGAGTTAAGGCTATGAAGTTCCC ATTGAAGACATCTGAAAGAAAGGAAGAAAGAGAAGCAGTTGAA GCTGCATAA

**Table A2:** Primers used in this study

Primer	Sequence
UP‐F	GCGGAGAAGTCGTTGATAGCA
DOWN‐R	GATCATAGATCCGGCACTTAGAG
Hl‐UP(40)‐R	GAAGAAGTATGTAACAACATTTTTTTACGTATCGCTTTGTTTT
Hl‐ZEP(40)‐F	ACAAAGCGATACGTAAAAAAATGTTGTTACATACTTCTTCATTG
Hl‐ZEP(40)‐R	AGCGGATGAATGCACGCGATTTAAGCTGCTTGAACCATTG
Hl‐DOWN(40)‐F	CAATGGTTCAAGCAGCTTAAATCGCGTGCATTCATCC
Hl‐UP(60)‐R	TCTTGGCAATGAAGAAGTATGTAACAACATTTTTTTACGTATCGCTTTGTTTT
Hl‐ZEP(60)‐F	CAAATATAAAACAAAGCGATACGTAAAAAAATGTTGTTACATACTTCTTCATTG
Hl‐ZEP(60)‐R	TTTCGGTTAGAGCGGATGAATGCACGCGATTTAAGCTGCTTGAACCATTG
Hl‐DOWN(60)‐F	AATAGAGGTGCAATGGTTCAAGCAGCTTAAATCGCGTGCATTCATCC
Hl‐UP(100)‐R	ACATGACCTGCAGCTTGACATCTTGGCAATGAAGAAGTATGTAACAACATTTTTTTACGTATCGCTTTGTTTT
Hl‐ZEP(100)‐F	TAATTATCTACTTTTTACAACAAATATAAAACAAAGCGATACGTAAAAAAATGTTGTTACATACTTCTTCATTG
Hl‐ZEP(100)‐R	AGGTTGTCTAACTCCTTCCTTTTCGGTTAGAGCGGATGAATGCACGCGATTTAAGCTGCTTGAACCATTG
Hl‐DOWN(100)‐F	GTGACTGTTACCAAAGAATTAATAGAGGTGCAATGGTTCAAGCAGCTTAAATCGCGTGCATTCATCC
Sl‐UP(40)‐R	TAGAAAACAGTAGAGTACATTTTTTTACGTATCGCTTTGTTT
Sl‐ZEP(40)‐F	ACAAAGCGATACGTAAAAAAATGTACTCTACTGTTTTCTATACAT
Sl‐ZEP(40)‐R	AGCGGATGAATGCACGCGATTTATGCAGCTTCAACTGCT
Sl‐DOWN(40)‐F	AAGCAGTTGAAGCTGCATAAATCGCGTGCATTCATCC
Sl‐UP(60)‐R	AACTGATGTATAGAAAACAGTAGAGTACATTTTTTTACGTATCGCTTTGTTTT
Sl‐ZEP(60)‐F	CAAATATAAAACAAAGCGATACGTAAAAAAATGTACTCTACTGTTTTCTATACAT
Sl‐ZEP(60)‐R	TTTCGGTTAGAGCGGATGAATGCACGCGATTTATGCAGCTTCAACTGCT
Sl‐DOWN(60)‐F	GAAGAAAGAGAAGCAGTTGAAGCTGCATAAATCGCGTGCATTCATCC
Sl‐UP(100)‐R	AAAACTGAAGTAGATGGATGAACTGATGTATAGAAAACAGTAGAGTACATTTTTTTACGTATCGCTTTGTTTT
Sl‐ZEP(100)‐F	TAATTATCTACTTTTTACAACAAATATAAAACAAAGCGATACGTAAAAAAATGTACTCTACTGTTTTCTATACAT
Sl‐ZEP(100)‐R	AGGTTGTCTAACTCCTTCCTTTTCGGTTAGAGCGGATGAATGCACGCGATTTATGCAGCTTCAACTGCT
Sl‐DOWN(100)‐F	TGAAGACATCTGAAAGAAAGGAAGAAAGAGAAGCAGTTGAAGCTGCATAAATCGCGTGCATTCATCC
F1‐F	GCGGAGAAGTCGTTGATAGCA
F3‐R	GATCATAGATCCGGCACTTAGAG
Hl‐F1‐R	AACATTTTTTTACGTATCGCTTTGTTTTAT
Hl‐F2‐F	ATAAAACAAAGCGATACGTAAAAAAATGTT
Hl‐F2‐R	GAATGCACGCGATTTAAGC
Hl‐F3‐F	GCTTAAATCGCGTGCATTCAT
Sl‐F1‐R	AGTAGAGTACATTTTTTTACGTATCGC
Sl‐F2‐F	GCGATACGTAAAAAAATGTACTCTACT
Sl‐F2‐R	GATGAATGCACGCGATTTATG
Sl‐F3‐F	GCATAAATCGCGTGCATTCAT
